# Comparison of the Clinical Value of the Geriatric Nutritional Risk Index and Prognostic Nutritional Index as Determinants of Survival Outcome in Patients with Gastric Cancer

**DOI:** 10.7150/jca.77397

**Published:** 2022-09-21

**Authors:** Soomin An, Wankyu Eo, Sookyung Lee

**Affiliations:** 1 Department of Nursing, Dongyang University, Gyeongbuk, Republic of Korea; 2 College of Medicine, Kyung Hee University, Seoul, Republic of Korea; 3 Department of Clinical Oncology, College of Korean Medicine, Kyung Hee University, Seoul, Republic of Korea

**Keywords:** Gastrectomy, Nutritional indices, Prognosis, Stomach neoplasm

## Abstract

**Background:** The geriatric nutritional risk index (GNRI) is an important determinant of overall survival (OS) in patients with stage I-III gastric cancer (GC) across all ages; however, its value as a determinant of disease-free survival (DFS) is unclear. Moreover, the prognostic values between the GNRI and prognostic nutritional index (PNI) remains unclear.

**Methods:** We retrospectively evaluated the value of the GNRI and PNI as determinants of OS and DFS in patients with stage I-III GC who underwent curative-intent gastrectomy. Cox regression analysis was used for evaluating the determinants of survival outcomes. The discriminative capacity of the prognostic model was determined using the concordance index (C-index), and then C-indices of related models were compared.

**Results:** Data from 450 patients were analyzed. The median patient age was 60 years (range: 26-92 years). In total, 276 (61.3%) patients had stage I cancer, 83 (18.4%) had stage II cancer, and 91 (20.2%) had stage III cancer. Multivariate Cox regression analysis revealed that age, type of gastrectomy (TOG), T stage, tumor-node-metastasis (TNM) stage, and GNRI were determinants of OS. These five covariates constituted the GNRI model for the OS. In addition, multivariate analysis revealed that age, TOG, TNM stage, and GNRI were determinants of DFS. These four covariates constituted the GNRI model for DFS. When constructing the PNI model for OS (comprising age, TOG, T stage, TNM stage, and PNI), and PNI model for DFS (including age, TOG, TNM stage, and PNI), the C-indices of the GNRI and PNI models were nearly equal for OS (0.818 and 0.818, respectively; *p*=0.909) and DFS (0.805 and 0.808, respectively; *p*=0.653). Using the GNRI models, nomograms for predicting OS and DFS were established. When validating the nomograms using calibration curves, the predicted survival closely matched the actual survival rate.

**Conclusion**: The GNRI and PNI were important determinants of both OS and DFS in patients with GC across all ages. In addition, the effects of the GNRI model on OS and DFS were similar to those of the PNI model.

## Introduction

Gastrectomy is the standard treatment for gastric cancer (GC); however, substantial relapse and death can occur. Therefore, establishing biomarkers that accurately predict survival outcomes may help improve survival outcomes by providing useful information to clinicians before and after surgery.

The tumor-node-metastasis (TNM) staging system is regarded as the standard for predicting prognosis in patients with cancer; however, it has disadvantages, such as a differing prognosis for the same TNM stage 1, 2. Inflammatory markers, including the absolute monocyte and lymphocyte count prognostic score 3, lymphocyte-to-monocyte ratio (LMR) 4, neutrophil-to-lymphocyte ratio (NLR) 5, and platelet-to-lymphocyte ratio (PLR) 5 have been reported as determinants of survival outcomes. Nonetheless, there is no consensus on the optimal cutoff points, thus, limiting its clinical use. Recently, measurement of minimal residual disease (MRD) following curative intent therapy has gained attention. Detection of MRD by measuring circulating tumor DNA (ctDNA) levels may facilitate individualized adjuvant therapy and improve survival outcomes in patients with GC. However, data on the clinical use of ctDNA in GC are limited, requiring further studies for its clinical application 6. Given the dissatisfaction with the established biomarkers, further research to develop accurate and novel biomarkers are necessary.

Malnutrition promotes tumor recurrence through tumor immunosuppression and is associated with poor survival outcomes 7. However, the gold standard for evaluating nutritional risks remains unclear 8. The prognostic nutritional index (PNI), which comprises the serum albumin level (ALB) and absolute lymphocyte count (ALC), is considered as an indicator of nutrition. The association between low PNI and poor overall survival (OS) has been reported in patients with various malignant tumors, including GC 7, 9, 10. The geriatric nutritional risk index (GNRI), which is composed of ALB, body weight, and height, is a nutritional index for survival outcomes in older patients with various malignancies 11. Reportedly, low GNRI is associated with poor survival outcomes in patients with various malignant tumors 8. Regarding patients with stage I-III GC, the GNRI is reported as a determinant of OS not only in patients aged >65 years 12 and >75 years 13 but also in patients of all age groups 14, 15. Thus, GNRI could be an important determinant of OS in patients with stage I-III GC across all ages; however, its value as a determinant of disease-free survival (DFS) is unclear. Moreover, the prognostic values between the GNRI and PNI remains unclear.

Therefore, the present study aimed to evaluate the clinical significance of the GNRI as a determinant of OS and DFS in patients with stage I-III GC across all ages. In addition, the prognostic values between the GNRI and PNI were compared. Further, in contrast to previous studies, the GNRI and PNI were treated as continuous variables without dichotomy to avoid potential bias.

## Methods

### Patients

Patients who underwent curative-intent gastrectomy at Kyung Hee University Hospital at Gangdong between June 2006 and December 2017 were analyzed. The inclusion criteria were as follows: (i) primary GC, (ii) stage I-III GC according to the American Joint Committee on Cancer staging system (8th edition) 16, and (iii) negative resection margins. The exclusion criteria included: (i) concurrent malignancies or malignancies within the past 5 years, (ii) administration of any anticancer treatment prior to surgery, (iii) development of severe infections within 4 weeks before gastrectomy, and (iv) pre-existing active infection or autoimmune diseases.

This study was approved by the Institutional Review Board of Kyung Hee University Hospital at Gangdong (2022-07014). The requirement for informed consent was waived owing to the retrospective design of the study.

### Baseline clinical characteristics

Data on clinicopathological parameters, including age, sex, body mass index (BMI), tumor site, type of gastrectomy (TOG), tumor size, T stage, nodal invasion, TNM stage, vascular invasion, histological classification based on Lauren's criteria 17, leukocyte count, ALC, absolute monocyte count (AMC), absolute neutrophil count (ANC), hemoglobin level (Hb), platelet count, and ALB, were analyzed. The LMR, NLR, and PLR were calculated according to formulas using preoperative blood samples obtained within 1 week before the surgery.

### Measurement of PNI and GNRI

The PNI was calculated as: PNI = 10 × ALB (g/dL) + 0.005 × ALC (per μL) 10. The GNRI calculation formula was as follows: GNRI = 14.89 × ALB (g/dL) + 41.7 × (current body weight [kg] / 22 × height [m]^2^). If (current body weight [kg] / 22 × height [m]^2^) >1, it was set to 1 11.

### Statistical analysis

Clinicopathological parameters, which are continuous variables, were expressed as medians with interquartile ranges (IQRs) in parentheses. OS was defined as the interval between the date of gastrectomy and date of death from any cause. DFS was defined as the interval between the date of gastrectomy and date of recurrence or death from any cause, whichever occurred first. The correlation between the GNRI and the clinicopathological parameters represented as continuous variables (i.e., age, BMI, tumor size, leukocyte count, ALC, AMC, ANC, Hb, platelet count, LMR, NLR, PLR, and PNI) was determined using Pearson's correlation coefficient. To facilitate he interpretation of correlations, a correlation matrix was formed. Nonparametric tests, such as the Kruskal-Wallis test or Mann-Whitney U test were used for between-group comparisons of categorical variables (age, sex, TOG, T stage, nodal invasion, TNM stage, vascular invasion, and histology). The Bonferroni method was used for multiple comparisons.

Hazard ratios (HRs) for continuous and categorical variables were determined using the Cox regression analysis. In this study, age, sex, BMI, TOG, tumor size, T stage, nodal invasion, TNM stage, vascular invasion, histology, anemia (Hb <13 g/dL in male patients and Hb <12 g/dL in female patients), LMR, NLR, PLR, GNRI, and PNI were analyzed. Multivariate Cox regression analysis was performed using the significant variables (*p*<0.05) in univariate Cox regression analysis. Multicollinearity in the variables was determined by calculating the variance inflation factor (VIF).

Furthermore, the discriminative capacity of the models was determined using the concordance index (C-index). The two C-indices were compared as described by Kang et al. 18. In addition, the C-index for OS and DFS of the models over 10 years was plotted using bootstrap cross-validation with 1,000 resamples replacing the original datasets.

Finally, nomograms for predicting OS and DFS were constructed using the established models and internally validated using calibration curves.

All *p*-values presented were two-sided, and statistical significance was set at *p*<0.05. Statistical analyses were performed using the R packages (r-project.org).

## Results

### Patients' clinical characteristics

The median patient age was 60 years (range: 26-92 years), and the median tumor size was 3.0 cm. While 354 (78.7%) patients underwent partial gastrectomy, 96 (21.3%) underwent a total gastrectomy. Regarding T stage, 259 (57.6%) patients had T1, 45 (10.0%) had T2, 99 (22.0%) had T3, and 47 (10.4%) had T4 invasion. In total, 276 (61.3%) patients had stage I cancer, 83 (18.4%) had stage II cancer, and 91 (20.2%) had stage III cancer. The median GNRI and PNI values were 102.8 and 51.3, respectively (Table 1).

### Correlation between GNRIs and clinicopathological parameters

**Figure 1:**
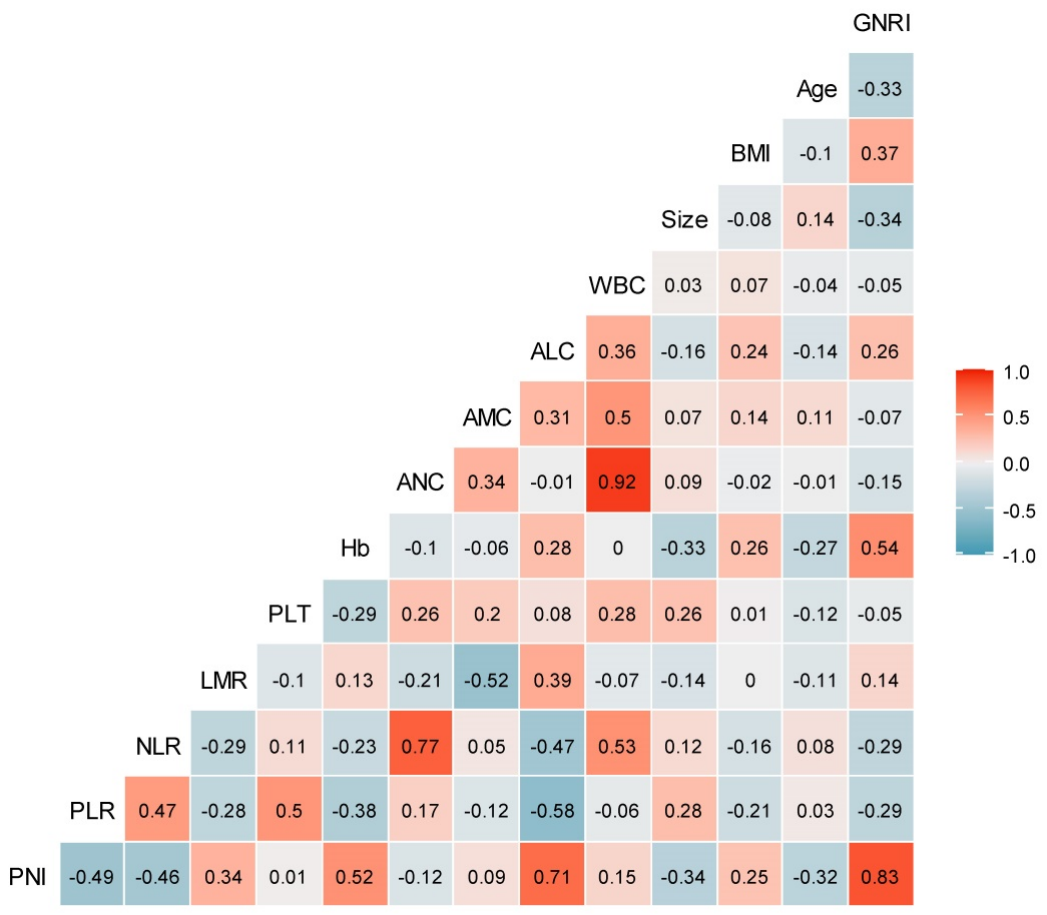
** Correlation coefficients between clinicopathological variables.** The number in the box represents the correlation coefficient (*r*). ALC: absolute lymphocyte count; AMC: absolute monocyte count; ANC: absolute neutrophil count; BMI: body mass index; GNRI: geriatric nutritional risk index; Hb: hemoglobin level; LMR: lymphocyte-to-monocyte ratio; NLR: neutrophil-to-lymphocyte ratio; PLR: platelet-to-lymphocyte ratio; PLT: platelet count; PNI: prognostic nutritional index; WBC: white blood cell.

No significant correlation was found between GNRIs and most continuous variables (such as age, BMI, tumor size, leukocyte count, Hb, platelet count, ALC, AMC, ANC, LMR, NLR, and PLR) in Pearson's correlation coefficient analysis. However, a significant correlation was noted between the GNRI and PNI (*r*=0.83) (Fig. 1).

Moreover, no significant difference was observed in the GNRIs between the groups for categorical variables, such as sex and histology. However, there were significant differences in GNRI between the groups for variables (such as age, TOG, T stage, nodal invasion, TNM stage, and vascular invasion) (Table 2). In multiple comparisons, there were significant differences in GNRIs between stage I and stage II (*p*=0.010) as well as between stage I and stage III cancers (*p*<0.001); however, no significant difference existed in the GNRIs between stage II and stage III cancers (*p*=0.140).

### Cox regression of the risk factors of OS and DFS

The median and IQR of the follow-up time was 72.0 months (28.4-97.3 months). Regarding OS, variables such as age, TOG, tumor size, T stage, nodal invasion, TNM stage, vascular invasion, anemia, LMR, NLR, PLR, GNRI, and PNI were significant in univariate Cox regression analysis. Multivariate Cox regression analysis, excluding PNI, revealed that age (hazard ratio [HR] 1.05, *p*<0.001), TOG (HR 1.86, *p*=0.004), T stage (HR 1.96, *p*=0.025), TNM stage (HR 2.44, *p*=0.002), and GNRI (HR 0.94, *p*<0.001) were significant variables, and VIFs were 1.04, 1.03, 1.92, 1.88, and 1.09, respectively. Meanwhile, excluding the GNRI instead of the PNI, the significant variables were age (HR 1.05, *p*<0.001), TOG (HR 1.95, *p*=0.002), T stage (HR 1.90, *p*=0.032), TNM stage (HR 2.47, *p*=0.001), and PNI (HR 0.92, *p*<0.001). The VIFs were 1.06, 1.03, 1.90, 1.85, and 1.11, respectively (Table 3).

Using the univariate Cox model, the same variables as those for OS were identified as the significant determinants of DFS. On multivariate Cox regression analysis excluding PNI, the significant determinants of DFS were age (HR 1.04, *p*<0.001), TOG (HR 1.86, *p*=0.004), TNM stage (HR 4.18, *p*<0.001), and GNRI (HR 0.94, *p*<0.001), and the VIFs were 1.08, 1.01, 1.07, and 1.12, respectively. Furthermore, after excluding the GNRI instead of the PNI, the significant variables were age (HR 1.04, *p*<0.001), TOG (HR 1.91, *p*=0.002), TNM stage (HR 4.16, *p*<0.001), and PNI (HR 0.92, *p*<0.001), and their VIFs were 1.08, 1.02, 1.06, and 1.12, respectively (Table 4).

### Establishment and validation of prognostic models

The four variables (i.e., age, TOG, T stage, and TNM stage) constituted the baseline model for OS. The GNRI and PNI models for OS were constructed by adding GNRI and PNI to the baseline variables, respectively. The three variables (i.e., age, TOG, and TNM stage) constituted the baseline model for DFS. The GNRI and PNI models for DFS were constructed by adding GNRI and PNI to the baseline variables, respectively.

**Figure 2:**
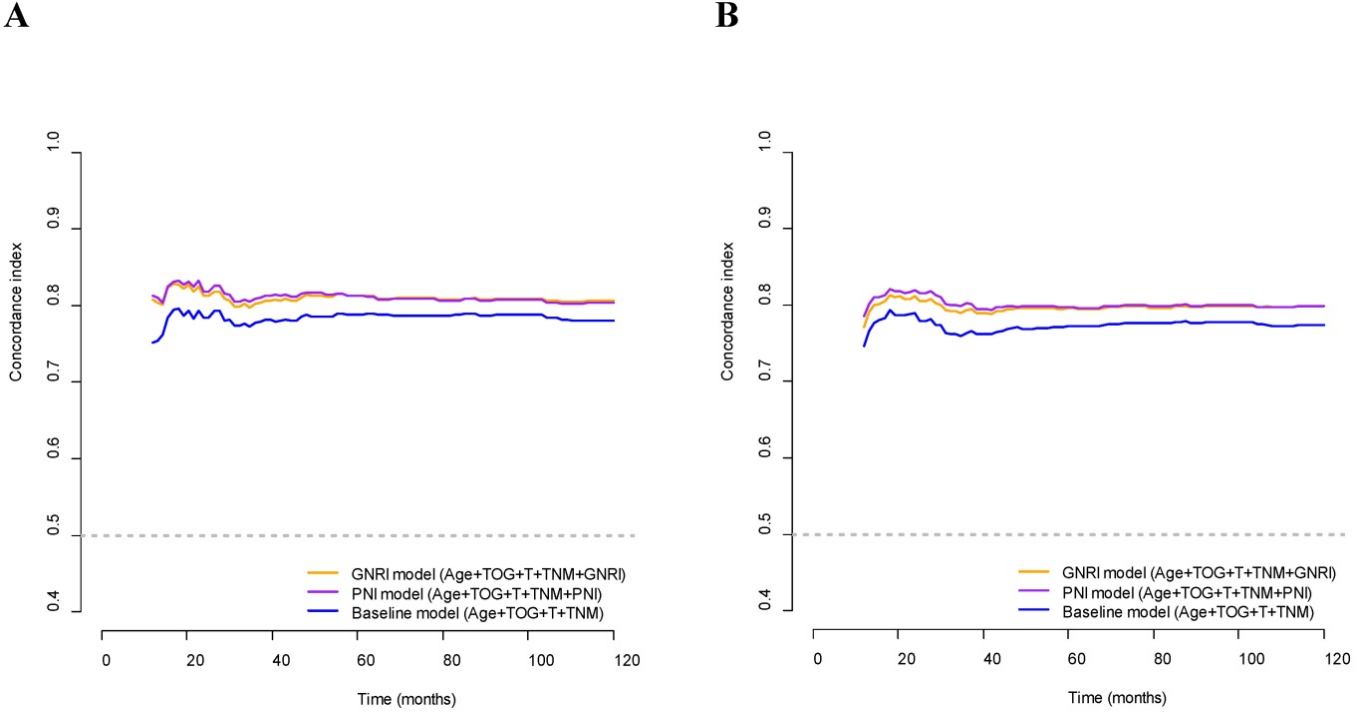
** Concordance indices of GNRI, PNI, and baseline models for survival outcomes.** (A) Overall survival; (B) Disease-free survival. GNRI: geriatric nutritional risk index; PNI: prognostic nutritional index; T: T stage; TNM: tumor-node-metastasis stage; TOG: type of gastrectomy

The C-index of the GNRI model was significantly higher than that of the baseline model for OS (0.818 and 0.794, respectively; *p*<0.001) and DFS (0.805 and 0.781, respectively; *p*=0.013). In addition, the C-indices of the PNI model were significantly higher than those of the baseline model for OS (0.819 and 0.794, respectively; *p*=0.039) and DFS (0.808 and 0.781, respectively; *p*=0.025). The C-indices of the GNRI and PNI models for OS and DFS were higher than those of the respective baseline models for OS and DFS over the 10 years (Fig. 2). On comparing the GNRI model with the PNI model, the C-index of the GNRI model was nearly equal to that of the PNI model for OS (0.818 and 0.818, respectively; *p*=0.909) and DFS (0.805 and 0.808, respectively; *p*=0.653). The C-indices of the GNRI model for OS and DFS were comparable to those of the PNI model over the 10 years (Fig. 2).

**Figure 3:**
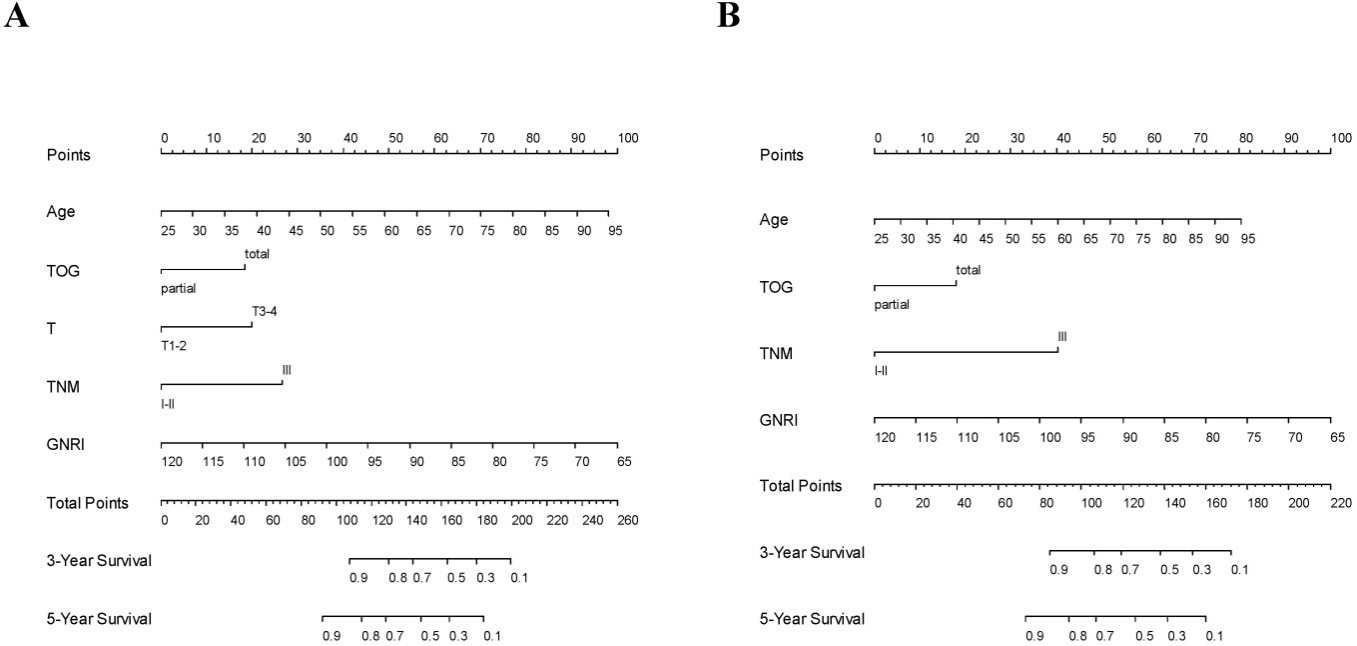
** Nomograms predicting 3-year and 5-year survival.** (A) Overall survival; (B) Disease-free survival. GNRI: geriatric nutritional risk index; T: T stage; TNM: tumor-node-metastasis stage; TOG: type of gastrectomy

**Figure 4:**
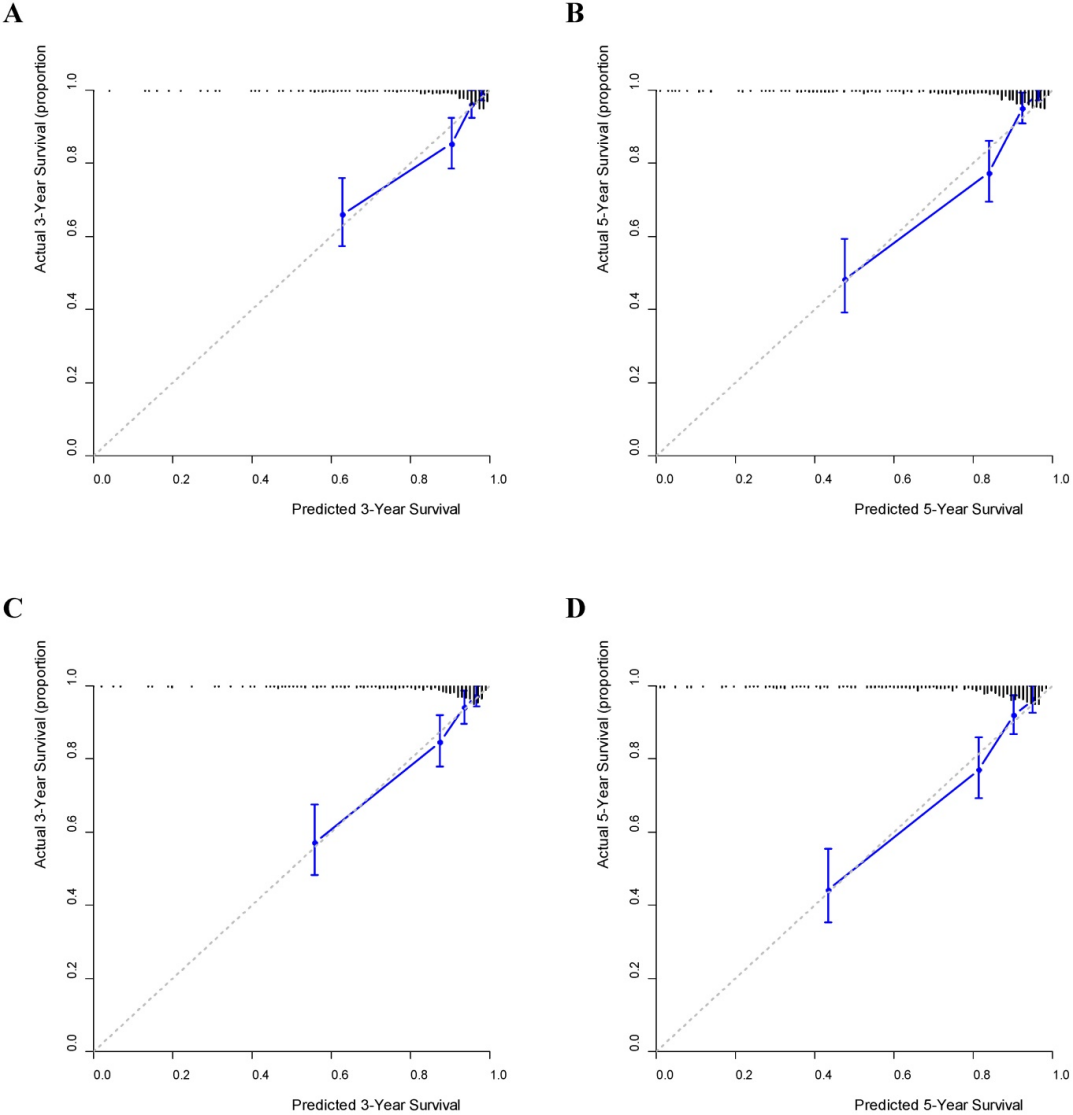
** Calibration curves predicting survival.** (A) 3-year overall survival (OS); (B) 5-year OS; (C) 3-year disease-free survival (DFS); (D) 5-year DFS.

Using the GNRI model, nomograms for predicting the OS and DFS were established (Fig. 3). Furthermore, on validating the nomograms using calibration curves, the predicted survival closely matched the actual survival (Fig. 4).

## Discussion

This study evaluated the prognostic potential of the GNRI and PNI in patients with stage I-III GC and found that the GNRI and PNI were determinants of both OS and DFS. Additionally, the effects of the GNRI model on DFS and OS were similar to those of the PNI model.

In this study, the GNRI was evaluated as a continuous variable instead of a categorical variable because the optimal cutoff value obtained by minimizing the *p*-value is prone to bias and has limited application in other cohorts 19. There was no significant correlation between the GNRI and clinicopathological parameters (such as age, BMI, tumor size, leukocyte count, Hb, platelet count, LMR, NLR, PLR, and PNI). Regarding age, the results of this study are inconsistent with those of previous studies, which showed a significant correlation between GNRI and age 14, 15. However, there was a significant difference in the median GNRI values between the age groups (104.0 in patients aged <65 years, 99.8 in ≥ 65 years; *p*<0.001). Therefore, discrepancies in the results between studies may result from differences in the treatment of age as a continuous or categorical variable.

In addition, significant differences in GNRI were observed for pathological variables (e.g., T stage, nodal invasion, TNM stage, and vascular invasion). These results are concurrent with those of previous studies, which showed a significant correlation between the GNRI and T stage, nodal invasion, and TNM stage 13-15. This finding suggests that GNRI may be affected by the extent of tumor invasion.

In this study, multivariate Cox regression analysis showed that GNRI was a determinant of OS in GC (HR 0.94, *p*<0.001). Similarly, previous studies have found that the GNRI is a prognostic factor for OS in patients with GC 12-15. Moreover, we found that the GNRI was a determinant of DFS in patients with GC (HR 0.94, *p*<0.001). However, no available studies have evaluated the clinical role of the GNRI as a determinant of DFS; hence, future studies validating this are required.

Better survival outcomes in patients with higher GNRIs have been reported in various types of solid tumors (e.g., lung cancer, hepatoma, esophageal cancer, kidney cancer, prostate cancer, and diffuse large B-cell lymphoma) 8, 20. However, the underlying mechanism that enables GNRI to determine survival outcomes has not been completely elucidated. BMI, a major component of the GNRI, is considered a determinant of survival, and a low BMI before surgery indicates poor prognosis 21. ALB, another major component of the GNRI, is an indicator of nutritional status and systemic inflammatory responses 22-26. Albumin synthesis is attenuated by tumor necrosis factor-α or interleukin-6 27. Decreased ALB in patients with GC is associated with an increased risk of postoperative infectious complications and worse survival outcomes 2, 25, 28-30. Therefore, the clinical value of the GNRI in determining survival outcomes may be attributed to the synergistic effects of its two major components—BMI and ALB.

On comparing the baseline and GNRI models, the C-indices for OS and DFS were significantly higher in the GNRI model than in the baseline model (*p*<0.001 for OS and *p*=0.013 for DFS). This finding suggested that the GNRIs have clinical value in determining survival outcomes. Using the GNRI model, we established nomograms to predict the 3-year and 5-year OS and DFS rates and verified the nomograms using calibration curves. Together with age, the GNRI accounted for the main component of the overall scores in the nomogram, thus, indicating the clinical value of GNRIs as predictors of survival.

The PNI, which consists of ALB and ALC, has been considered as a determinant of both OS and DFS in GC 10, 31. In the present study, PNI was a determinant of OS (HR 0.92, *p*<0.001) and DFS (HR 0.92, *p*<0.001) in the multivariate Cox regression analysis. Therefore, this finding is consistent with those of previous studies. On comparing the baseline and PNI models, the C-indices for OS and DFS were significantly higher in the PNI model than in the baseline model (*p*=0.039 for OS and *p*=0.025 for DFS). This finding suggests that PNIs have a clinical value in determining survival outcomes.

The underlying mechanism, which enables PNI to determine survival outcomes, has not been completely elucidated. ALC is a major component of the PNI. A decrease in ALC, as seen in malignant tumors, potentially reflects an insufficient response of the host immune system to tumors, consequently enhancing tumor progression 32-36. In addition, ALC is considered a marker of nutritional status 26. ALB, another major component of the PNI, is considered an indicator of nutritional status and systemic inflammatory responses 22-26, and patients with decreased ALB levels experience adverse survival outcomes 2, 25, 28-30. Therefore, the clinical value of the PNI in determining survival outcomes may be attributed to the synergistic effects of its two major components—ALC and ALB.

In this study, in addition to the GNRI and PNI, age, TOG, T stage, and TNM stage were found to be determinants of OS, while age, TOG, and TNM stage were found to be determinants of DFS. The prognostic value of age and TNM stage as determinants of survival in patients with GC has been reported previously 12, 14, 15, 37. Regarding TOG, thirty-day morbidity after gastrectomy and readmission rates due to nutritional difficulties were high in patients undergoing total gastrectomy (TG) 38, 39. Additionally, TG is a determinant of OS and cancer-specific survival (CSS) 14. Therefore, the results of the present study are consistent with those of the previous studies. Regarding T stage, Matsunaga et al. showed that T stage was a determinant of CSS, but not OS, in a multivariate analysis 13. However, in the study by Tonello et al., T stage was a determinant of OS in the multivariate analysis, and the results are compatible with those of the present study 40.

The gold standard for evaluating nutritional risk remains unclear 8. Therefore, in this study, the clinical value of the GNRI was compared to that of the PNI. Considering the highly significant correlation between the GNRI and PNI (*r*=0.83), their clinical significance was not analyzed in the same model but in separate models (i.e., GNRI and PNI models). A comparison between the GNRI and PNI models revealed that the C-indices of the two models were nearly equal for OS (0.818 and 0.818, respectively; *p*=0.909) and DFS (0.805 and 0.808, respectively; *p*=0.653). Therefore, the GNRI is equivalent to the PNI in terms of nutritional markers for survival outcomes. However, there are no available studies evaluating the clinical role of the GNRI versus PNI as determinants of OS and DFS, and future studies are needed to validate this.

The strengths of this study are as follows: First, the GNRI and PNI were important determinants of both OS and DFS in patients with GC without age restriction when used as continuous variables. To the best of our knowledge, the value of the GNRI as a determinant of DFS has not yet been reported in patients with stage I-III GC. Second, there were significant differences in the GNRIs between the groups with respect to pathological variables (e.g., T stage, nodal invasion, TNM stage, and vascular invasion). This finding suggests that GNRI may be affected by the extent of tumor invasion. Third, the GNRI model was equivalent to the PNI model in terms of its ability to discriminate survival outcomes. Fourth, the predicted survival closely matched the actual survival rate when a prognostic nomogram was established using the GNRI model.

However, the present study has some limitations. First, because this was a retrospective study, limited survival outcome information for overseas patients (3.3% of the total) was inevitable, and this may have affected the results. Second, although potential bias was controlled, this was a single-center data analysis without external validation.

In conclusion, multivariate Cox regression analysis showed that the GNRI and PNI were prognostic factors for OS and DFS in patients with GC across all ages. The GNRI model has a higher ability to discriminate survival outcomes than the baseline model, and is nearly identical to the PNI model. The predicted survival closely matched the actual survival when nomograms were established using the GNRI model, implying that the GNRI model is a clinically significant predictor of survival in GC.

## Figures and Tables

**Table 1:** Patients' characteristics

Variables	Median (IQR) or *n* (%)
**Age, years**	60.0 (52.0-69.0)
**Sex**	
Male	301 (66.9%)
Female	149 (33.1%)
**BMI, kg/m^2^**	23.8 (21.4-26.0)
**Site of tumor**	
Upper	47 (10.5%)
Middle	154 (34.2%)
Lower	243 (54.0%)
Diffuse	6 (1.3%)
**Types of gastrectomy**	
Partial	354 (78.7%)
Total	96 (21.3%)
**Size of tumor, cm**	3.0 (2.0-5.5)
**T stage**	
T1	259 (57.6%)
T2	45 (10.0%)
T3	99 (22.0%)
T4	47 (10.4%)
**Nodal invasion**	
No	294 (65.3%)
Yes	156 (34.7%)
**TNM stage**	
I	276 (61.3%)
II	83 (18.5%)
III	91 (20.2%)
**Vascular invasion**	
No	430 (95.6%)
Yes	20 (4.4%)
**Histology (Lauren)**	
Intestinal	218 (48.4%)
Diffuse	108 (24.0%)
Mixed	105 (23.4%)
Unknown	19 (4.2%)
**Adjuvant chemotherapy**	
No	293 (65.1%)
Yes	157 (34.9%)
**Leukocyte, per μL**	6470 (5310-7700)
**ALC, per μL**	1927 (1550-2294)
**AMC, per μL**	461 (365-569)
**ANC, per μL**	3646 (2881-4730)
**Hemoglobin, g/dL**	13.1 (11.4-14.2)
**Platelet, ×10^3^/μL**	236 (203-278)
**Albumin, g/dL**	4.1 (3.9-4.3)
**LMR**	4.3 (3.3-5.4)
**NLR**	1.9 (1.4-2.6)
**PLR**	121.9 (96.4-157.6)
**PNI**	51.3 (47.3-54.6)
**GNRI**	102.8 (98.3-105.7)

ALC: absolute lymphocyte count; AMC: absolute monocyte count; ANC: absolute neutrophil count; BMI: body mass index; GNRI: geriatric nutritional risk index; IQR: interquartile range; LMR: lymphocyte-to-monocyte ratio; NLR: neutrophil-to-lymphocyte ratio; PLR: platelet-to-lymphocyte ratio; PNI: prognostic nutritional index; TNM: tumor-node-metastasis

**Table 2:** Geriatric nutritional risk indices in the categorical variables

Variables	GNRI	
	Median (IQR)	*p*-value
**Age**		
<65	104.0 (99.8-107.2)	<0.001
≥65	99.8 (95.4-104.2)	
**Sex**		
Male	102.8 (97.9-105.7)	0.708
Female	102.5 (98.4-105.7)	
**Types of gastrectomy**		
Partial	102.75 (98.4-105.7)	0.044
Total	100.4 (95.4-105.7)	
**T stage**		
T1	103.6 (99.8-105.7)	<0.001
T2	102.5 (99.8-107.2)	
T3	99.8 (95.1-104.2)	
T4	97.6 (90.5-102.8)	
**Nodal invasion**		
No	102.8 (99.8-105.7)	<0.001
Yes	100.1 (94.3-104.2)	
**TNM stage**		
I	102.8 (99.8-105.7)	<0.001
II	101.3 (96.8-105.7)	
III	99.8 (93.4-104.2)	
**Vascular invasion**		
No	102.8 (98.3-105.7)	0.010
Yes	97.1 (89.3-103.1)	
**Histology (Lauren)**		
Intestinal	102.5 (97.6-105.7)	0.295
Others	102.8 (98.5-105.7)	

GNRI: geriatric nutritional risk index; IQR: interquartile range; TNM: tumor-node-metastasis

**Table 3:** Univariate and multivariate Cox regression of overall survival

Covariate	Univariate analysis			GNRI model			PNI model	
	HR (95% CI)	*p*-value		HR (95% CI)	*p*-value		HR (95% CI)	*p*-value
Age, years†	1.06 (1.04-1.08)	<0.001		1.05 (1.03-1.07)	<0.001		1.05 (1.03-1.07)	<0.001
Sex (female vs male)	0.78 (0.50-1.20)	0.270						
BMI, kg/m^2^†	0.95 (0.87-1.03)	0.190						
TOG (total vs partial)	2.63 (1.74-3.98)	<0.001		1.86 (1.22-2.83)	0.004		1.95 (1.28-2.97)	0.002
Tumor size, cm†	1.19 (1.14-1.24)	<0.001						
T stage (T3-4 vs T1-2)	5.04 (3.01-7.68)	<0.001		1.96 (1.09-3.52)	0.025		1.90 (1.06-3.41)	0.032
Nodal invasion (yes vs no)	3.81 (2.53-5.74)	<0.001						
TNM stage (III vs I-II)	5.54 (3.71-8.28)	<0.001		2.44 (1.40-4.25)	0.002		2.47 (1.42-4.28)	0.001
Vascular invasion (yes vs no)	3.36 (1.74-6.47)	<0.001						
Histology (intestinal vs others)	0.89 (0.60-1.33)	0.578						
Anemia (yes vs no)‡	3.47 (2.31-5.22)	<0.001						
LMR†	0.80 (0.69-0.92)	0.002						
NLR†	1.17 (1.10-1.25)	<0.001						
PLR†	1.00 (1.00-1.00)	<0.001						
GNRI†	0.93 (0.91-0.94)	<0.001		0.94 (0.92-0.96)	<0.001			
PNI†	0.88 (0.85-0.90)	<0.001					0.92 (0.89-0.95)	<0.001

† Continuous variable; ‡ cutoff points are Hb <13 g/dL in men and Hb <12 g/dL in women. BMI: body mass index; CI: confidence interval; GNRI: geriatric nutritional risk index; HR: hazard ratio; LMR: lymphocyte-to-monocyte ratio; NLR: neutrophil-to-lymphocyte ratio; PLR: platelet-to-lymphocyte ratio; PNI: prognostic nutritional index; TNM: tumor-node-metastasis; TOG: type of gastrectomy

**Table 4:** Univariate and multivariate Cox regression of disease-free survival

Covariate	Univariate analysis			GNRI model			PNI model	
	HR (95% CI)	*p*-value		HR (95% CI)	*p*-value		HR (95% CI)	*p*-value
Age, years†	1.06 (1.04-1.07)	<0.001		1.04 (1.02-1.06)	<0.001		1.04 (1.02-1.06)	<0.001
Sex (female vs male)	0.67 (0.44-1.03)	0.070						
BMI, kg/m^2^†	0.95 (0.87-1.02)	0.164						
TOG (total vs partial)	2.44 (1.64-3.62)	<0.001		1.86 (1.22-2.83)	0.004		1.91 (1.28-2.86)	0.002
Tumor size, cm†	1.19 (1.14-1.23)	<0.001						
T stage (T3-4 vs T1-2)	4.72 (3.18-7.00)	<0.001						
Nodal invasion (yes vs no)	4.13 (2.79-6.12)	<0.001						
TNM stage (III vs I-II)	5.79 (3.95-8.49)	<0.001		4.18 (2.80-6.24)	<0.001		4.16 (2.79-6.21)	<0.001
Vascular invasion (yes vs no)	3.94 (2.16-7.19)	<0.001						
Histology (intestinal vs others)	0.93 (0.64-1.36)	0.707						
Anemia (yes vs no)‡	3.38 (2.30-4.97)	<0.001						
LMR†	0.76 (0.66-0.87)	<0.001						
NLR†	1.17 (1.10-1.24)	<0.001						
PLR†	1.00 (1.00-1.00)	<0.001						
GNRI†	0.93 (0.91-0.95)	<0.001		0.94 (0.92-0.96)	<0.001			
PNI†	0.88 (0.85-0.90)	<0.001					0.92 (0.89-0.95)	<0.001

† Continuous variable; ‡ cutoff points are Hb <13 g/dL in men and Hb <12 g/dL in women. BMI: body mass index; CI: confidence interval; GNRI: geriatric nutritional risk index; HR: hazard ratio; LMR: lymphocyte-to-monocyte ratio; NLR: neutrophil-to-lymphocyte ratio; PLR: platelet-to-lymphocyte ratio; PNI: prognostic nutritional index; TNM: tumor-node-metastasis; TOG: type of gastrectomy
